# The Advertisements of Endowed Hospitals

**Published:** 1887-04-09

**Authors:** 


### The Advertisements of Endowed Hospitals.

M. P. writes:—"The fact that one of the endowed hospitals is now appealing for funds is of necessity a drain upon the resources from which the voluntary hospitals obtain their support; and this, together with the other claims made upon the charitable in consequence of the Jubilee, will make it difficult to finance many voluntary charities this year. I see upon the platforms of suburban railway stations, posters appealing for subscriptions for Guy's Hospital. If these posters are not put up free, no voluntary hospital can compete with such an expenditure for advertising; and I think it is a question if this manner of obtaining funds is in accord with the general opinion as to the cost of raising money for charitable purposes. In the event of the £100,000 being forthcoming for Guy's, it will be interesting to know how much of it has been spent in advertising, etc., and especially to know the cost of raising the last £40,000. It is not difficult to get money for any such object as a hospital, if the expense incurred in obtaining it is not considered."

*** We have ascertained from Dr. Steele, the excellent Superintendent of Guy's Hospital, that the advertisements to which "M. P." refers are placed at nearly all the stations of the L. B. & S. C. and S. E. Railways, at the request of the directors of the Companies, and at no cost to Guy's Hospital. The Companies are under special obligation to the Hospital for the reception of most cases of accident occurring on their lines, and also for the treatment of their *employés* when disabled by illness. Apart from the local notices, the directors of the two railways have subscribed liberally in response to the appeal from the Governors.—[Ed. T. H.]

